# R-on-T Phenomenon Causing Cardiac Arrest in a Post-coronary Artery Bypass Graft (CABG) Patient

**DOI:** 10.7759/cureus.82515

**Published:** 2025-04-18

**Authors:** Oluwatosin Emehinola, Ruhma Ali, Asma Jamil, Vinesh Kumar, Abu Taha, Richard Miller, Claudia Komer

**Affiliations:** 1 Internal Medicine, New York Medical College (NYMC) at Saint Michael's Medical Center, Newark, USA; 2 Pulmonary and Critical Care Medicine, New York Medical College (NYMC) at Saint Michael's Medical Center, Newark, USA; 3 Internal Medicine, University of California, San Francisco, San Francisco, USA; 4 Anesthesiology, New York Medical College (NYMC) at Saint Michael's Medical Center, Newark, USA

**Keywords:** coronary artery bypass graft (cabg), ejection fraction (ef), electrocardiogram (ekg), premature ventricular contraction (pvc), ventricular fibrillation, ventricular tachycardia

## Abstract

Cardiac arrhythmias are common in post-coronary artery bypass graft (CABG) settings. It is a common practice to use temporary epicardial pacing wires at the end of cardiac surgery to prevent fatal arrhythmias (e.g., bradycardia,atrioventricular (AV) block, and asystole). It may also be used for sequential atrio-ventricular pacing for improved cardiac output in patients with poor ejection fraction. Epicardial wires are usually implanted around the right atrium and ventricle. Not every patient requires temporary epicardial pacing. However, certain risk factors may predispose patients to life-threatening AV blocks and ventricular tachyarrhythmias. These risk factors include advancing age, valvular surgery, poor left ventricular function, structural heart disease, diabetes mellitus, preoperative beta-blocker or digoxin use, and pre-existing history of arrhythmias. Only a handful of cases have been described in the literature where this seemingly lifesaving measure can trigger life-threatening events.

Here, we describe a case where epicardial pacing wires trigger ventricular arrhythmias due to improper sensing.

## Introduction

Temporary epicardial pacing is sometimes required after cardiac surgery to optimize hemodynamic status and prevent arrhythmias and sudden cardiac death [1]. However, temporary epicardial pacing carries its own risks, including infection, perforation, myocardial damage, disruption of coronary anastomosis, and R-on-T phenomenon. Herein, we present a case of R-on-T phenomenon leading to ventricular fibrillation (VF) that developed post-coronary artery bypass graft (CABG) due to undersensing of the epicardial pacing wires. This is an uncommon occurrence, posing significant difficulty for physicians to suspect and prevent. The R-on-T phenomenon is a type of premature ventricular contraction (PVC) that occurs when an electrical impulse stimulates a vulnerable ventricle during the descending phase of the T wave, a period of partial refractoriness. Although rare, this can result in ventricular arrhythmias, including polymorphic ventricular tachycardia (VT) or VF, which can lead to cardiac arrest. This phenomenon is particularly seen in patients with a low threshold for VF, including those with high risk of myocardial ischemia, electrolyte derangements, and early repolarization [1,2]. This case highlights the significance of evaluating the use of temporary epicardial pacing in the presence of high-frequency PVCs.

## Case presentation

A 58-year-old male with a past medical history of varicose veins was admitted with the chief complaint of back pain of two months duration. The back pain was episodic, radiating toward the right side of the chest and both arms, and progressively worsened, especially with exertion, and was relieved upon rest. Patient stated his predominant complaint was the back pain, more than the chest pain, only deciding to present to the emergency department (ED) after having an episode of cough with blood-tinged sputum. He denied any shortness of breath, dizziness, palpitations, syncope, or fever. His family history was significant for coronary artery disease in his brother and father, who passed away in their 40s. Around two years ago, he had a cardiac stress test that was negative.

On admission, he was afebrile with a temperature of 98.0ºF, blood pressure of 171/105 mmHg, a heart rate of 110 beats per minute, a respiratory rate of 20 breaths per minute, and saturating 100% on room air.

Physical examination revealed sinus tachycardia, regular rhythm with no murmurs, jugular venous distension, and 1+ bilateral lower extremity edema. There was no reproducible tenderness to palpation, and the pulmonary exam was unremarkable. Admission labs are outlined in Table 1.

**Table 1:** Laboratory values on admission BNP: B-type natriuretic peptide; BUN: blood urea nitrogen; GFR: glomerular filtration rate; HbA1c: glycated hemoglobin; HDL: high-density lipoprotein; LDL: low-density lipoprotein; TSH: thyroid-stimulating hormone; WBC: white blood count

Lab Test	Lab value	Reference value
WBC	10.6	4.4-11 × 10^9^/L
Hemoglobin	14.4	13.5-17.5 g/dL
Platelets	172	150-450 × 10^9^/L
Neutrophils	9.2	1.7-7.0 × 10^9^/L
Sodium	140	136-145 mmol/L
Potassium	4.4	3.5-5.5 mmol/L
Chloride	108	98-110 mmol/L
Bicarbonate	25	20-31 mmol/L
BUN	18	6-24 mg/dL
Creatinine	0.84	0.7-1.3 mg/dL
Glucose	166	70-140 mg/dL
Calcium	8.8	8.6-10.4 mg/dL
GFR	>90	>90 mL/minute/1.73 m^2^
Anion gap	7	7-13 mmol/L
Lactic acid	1.33	0-2 mmol/L
BNP	208	<100 pg/mL
Triglycerides	282	0-150 mg/dL
HDL	45	>40 mg/dL
LDL	126	0-100 mg/dL
HbA1c	5.9	4-5.6%
TSH	2.43	0.5-4 mIU/L
Procalcitonin	0.19	0-0.5 ng/mL
D-dimer	1869	0-500 ng/mL

**Figure 1:**
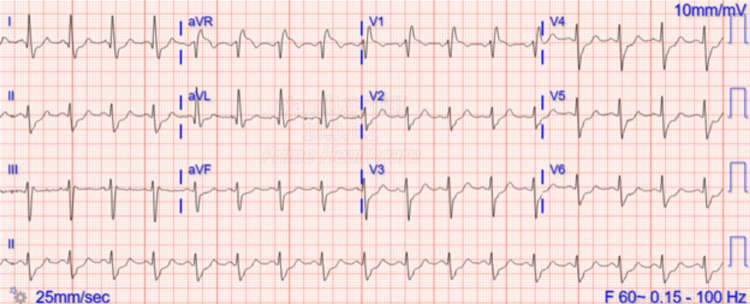
EKG on admission.

EKG on admission, as shown in Figure 1, revealed sinus tachycardia, ST depressions mainly in antero-lateral leads, right bundle branch block (RBBB), and left anterior fascicular block (LAFB) patterns.

High-sensitivity troponin I rose from 418 ng/L, peaking at 10804 ng/L (Table 2).

**Table 2:** Troponin trend within the first 12 hours of admission ABS: absolute, HS: high sensitivity

Time	20:54	00:15	03:32	05:47	09:24
HS troponin I	418	2100	6252	10535	10804
HS troponin I % change	-	402	298	26	3
HS troponin I ABS change	-	1682	6252	2183	269

**Figure 2:**
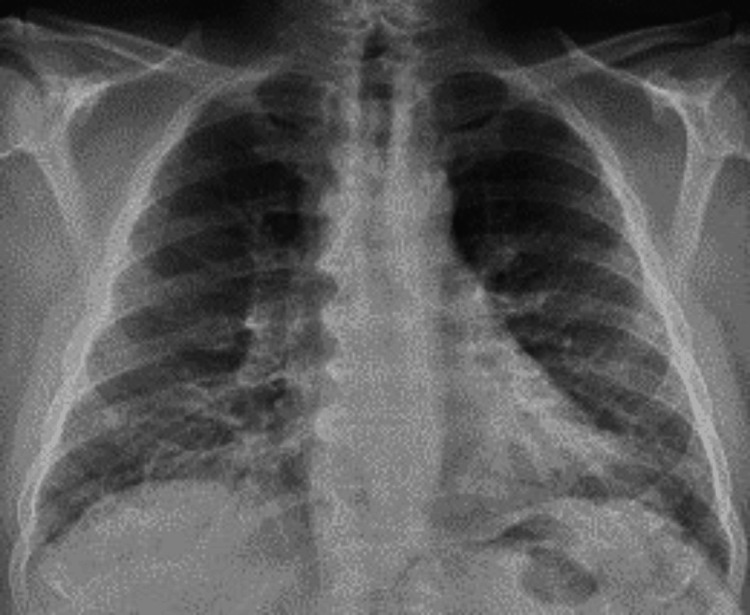
Chest X-ray showing patchy infiltrates in bilateral lower lobes.

**Figure 3:**
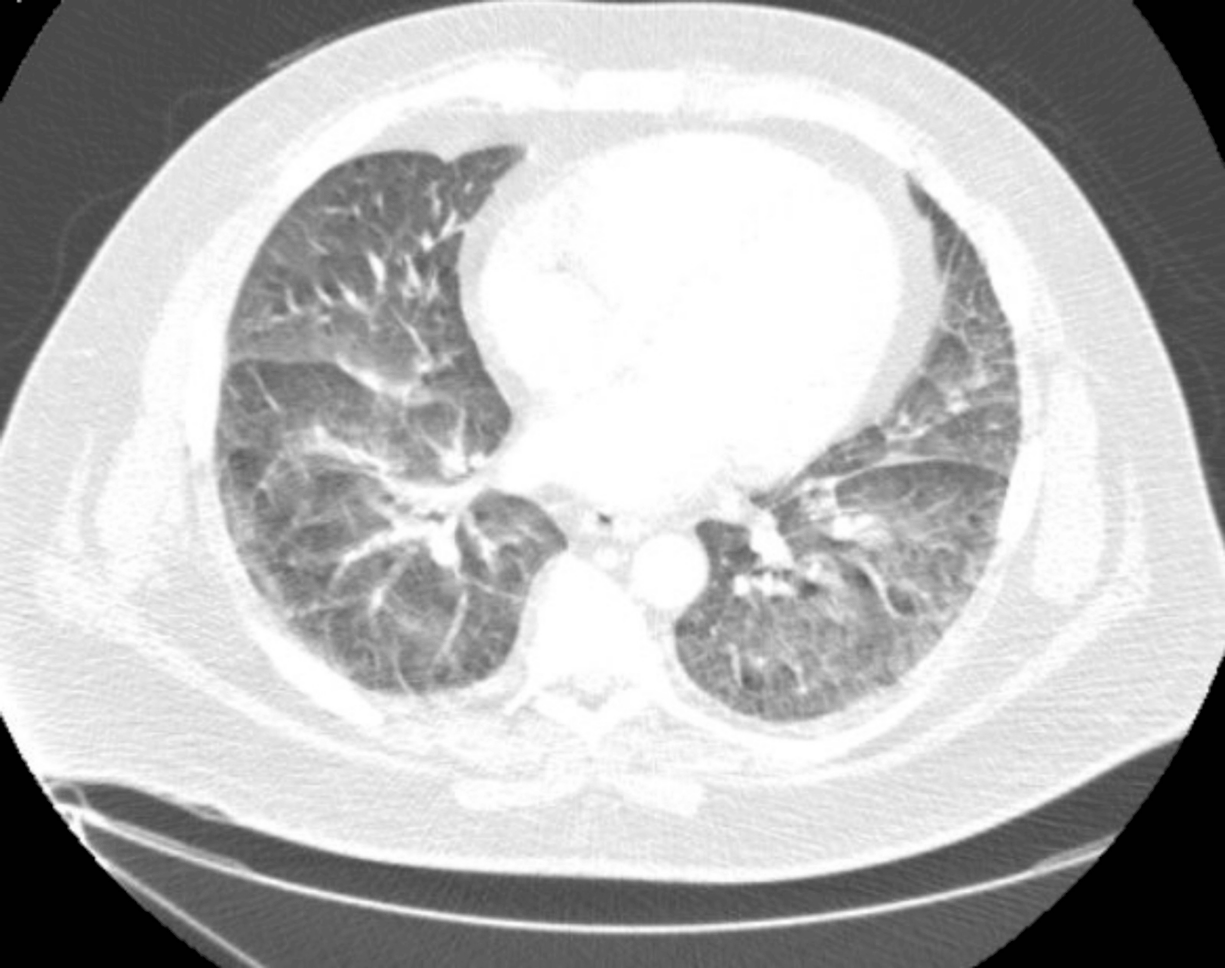
CT angiogram of chest showing diffuse pulmonary edema, ground glass opacities, mild bibasilar pleural effusions, and cardiomegaly.

The chest X-ray (Figure 2) showed bilateral patchy infiltrates in the lower lobes. The CT angiogram of the chest (Figure 3) on admission did not reveal aortic dissection, dilatation, or pulmonary embolism. However, it showed diffuse pulmonary edema with ground-glass opacities, mild bilateral basal pleural effusions, and cardiomegaly.

The echocardiogram showed reduced left ventricular systolic function with an ejection fraction (EF) of 30-35%. It also showed anteroseptal and anterior wall hypokinesis and a very small, focal apical aneurysm. There was no left ventricular thrombus. No previous echocardiograms were available in medical records to establish a prior baseline status.

**Figure 4:**
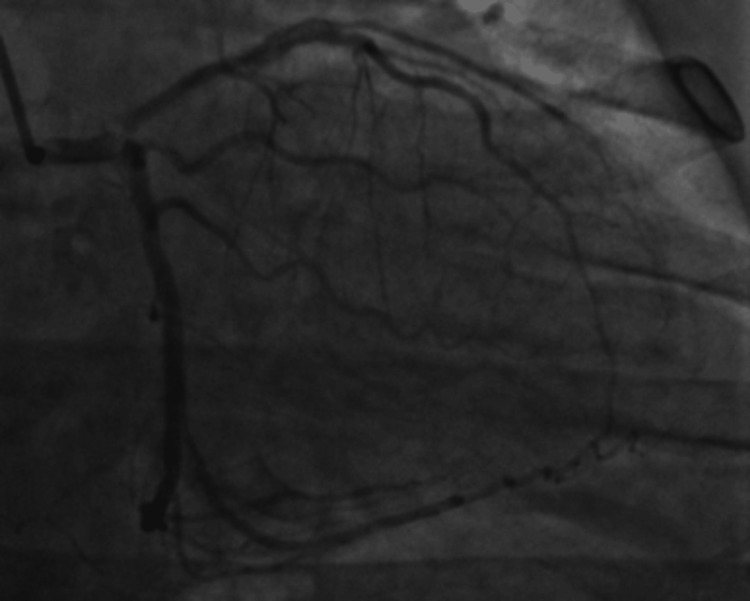
Coronary angiography showing LM bifurcating lesion and mid-LAD lesion. RCA circulation cannot be visualized. LAD: left anterior descending, LM: left main, RCA: right coronary artery

In the absence of very high-risk features, the patient was treated with medical therapy and planned for early invasive coronary angiography. The patient was loaded with aspirin, ticagrelor in the ED, started on heparin drip, and high-intensity atorvastatin. Coronary angiography (Figure 4) showed findings of the moderately calcified lesion in the distal left main (LM) extending to the ostium of the left anterior descending (LAD) and circumflex, with Medina classification of 1,1,1 and 70% diameter stenosis. This was the culprit lesion, and there was a thrombolysis in myocardial infarction (TIMI) flow of 2 across the lesion. There were also two non-culprit lesions, including a mid-LAD lesion with 50% diameter stenosis and TIMI flow of 2, and a proximal right coronary artery (RCA) lesion with 40% diameter stenosis and TIMI flow of 3. Cardiothoracic surgery was consulted in view of multivessel coronary artery disease (CAD) with LM involvement. The patient was subsequently planned for CABG.

The patient underwent a two-vessel bypass graft with left internal mammary artery (LIMA) to left LAD and saphenous vein graft for the obtuse marginal (OM) artery. There were no complications intraoperatively. EF on transesophageal echocardiogram (TEE) was 40% with no valvular abnormalities. The aortic cross-clamp time was 99 minutes, and cardiopulmonary bypass time was 137 minutes. Estimated blood loss was 500 mL.

In the immediate postoperative period, he required inodilator and vasopressor support with milrinone, levophed, and vasopressin. Blood sugars were tightly controlled per protocol with an insulin drip. Epicardial pacemakers were connected to the patient on VVI mode with a backup rate of 50. Patient cardiac index was four with slightly elevated pulmonary artery pressures. Due to decreased EF, the patient was kept intubated and sedated overnight and was extubated the next morning. The patient had an uneventful course on the morning following surgery. Telemetry showed non-sustained ventricular tachycardia (VTach) episodes that did not require any intervention. The patient’s electrolytes were kept above the goal of potassium more than 4 mg/dL and magnesium more than 2 mg/dL.

**Figure 5:**
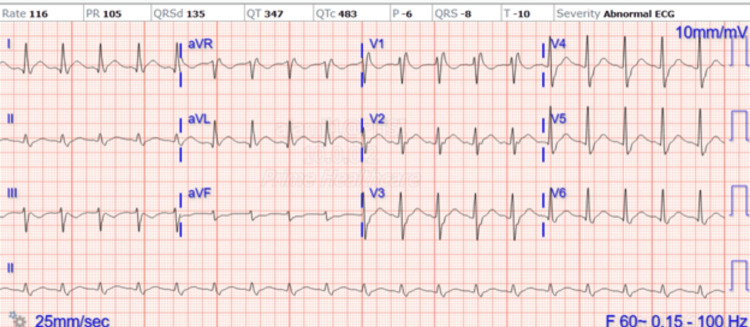
EKG post-cardiac arrest showing sinus tachycardia.

About 48 hours postoperatively, the patient was found to be in VTach and hypotensive while on a bilevel positive airway pressure (BiPAP) machine during sleep with oxygen saturation >94%. With the event, the patient became unresponsive, pulseless, and eventually desaturated. The Advanced Cardiovascular Life Support (ACLS) protocol was immediately started, and he was emergently re-intubated. One round of CPR was performed with one dose of epinephrine, and one defibrillation shock was delivered to the patient with return to sinus rhythm, as shown in Figure 5.

Arterial blood gas during the event on ventilator setting of AC/VC 100/500/20/5 showed mixed respiratory and metabolic acidosis with severe hypoxemia with improvement post return of spontaneous circulation (Table 3).

**Table 3:** Arterial blood gas during and post-cardiac arrest HCO_3_^-^: bicarbonate concentration, pCO_2_: Partial pressure of carbon dioxide, pO_2_: partial pressure of oxygen

Lab test	During cardiac arrest	Post-cardiac arrest (three hours)	Reference value
pH	7.16	7.44	7.34-7.44
pCO_2_	57.9	37	35-48 mmHg
pO_2_	60.8	116	75-100 mmHg
HCO_3_^-^	20.2	24	22-28 mmol/L

**Figure 6:**
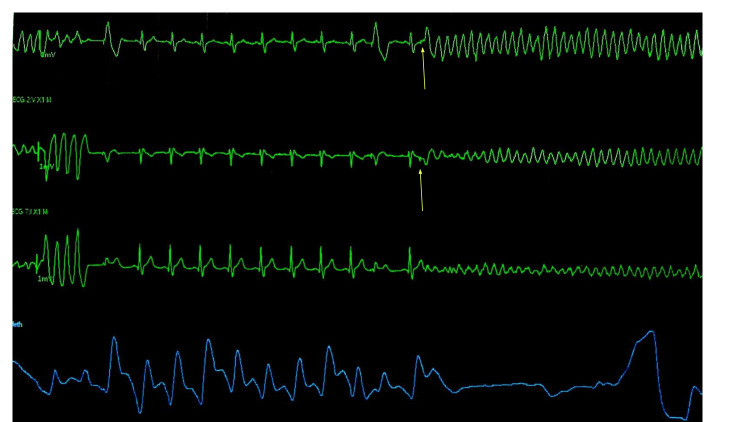
Cardiac telemetry showing polymorphic ventricular tachycardia devolving into ventricular fibrillation with R-on-T phenomenon as trigger.

Amiodarone bolus and infusion were initiated, while milrinone and low-dose norepinephrine infusions were also continued. Emergent TEE was performed to assess for new wall motion abnormalities given the concern of ischemia likely exacerbating VTach. TEE showed no new wall motion abnormalities, no pericardial effusion, an EF of 40-45%, and no valvular abnormalities. The TEE did not show findings suggestive of an ischemic event, and although the patient had moderate pulmonary hypertension, this rarely precipitates ventricular arrhythmias. Laboratory values during the event (Table 4) showed adequate electrolyte levels. A review of telemetry (Figure 6) during the event revealed polymorphic VTach preceding cardiac arrest with R-on-T wave as a trigger. Given the absence of electrolyte abnormalities, evidence for acute myocardial ischemia, and an unlikely pulmonary etiology, it was proposed that the epicardial pacemaker leads may have caused improper sensing and triggered the R-on-T phenomenon. A decision was made to turn off the epicardial pacemaker. No future arrhythmias were noted thereafter on telemetry. The patient was started on beta blockers, and he was extubated to a nasal cannula 24 hours later. Post-cardiac arrest echocardiogram suggested mildly reduced left ventricular systolic function with EF of 45-50% with anterolateral and anterior wall hypokinesis.

**Table 4:** Laboratory values during cardiac arrest ALP: alkaline phosphatase; ALT: alanine aminotransferase; AST: aspartate aminotransferase; BUN: blood urea nitrogen; WBC: white blood count

Lab test	Lab value	Reference value
WBC	20	4.4-11 × 10^9^/L
Hemoglobin	8.5	13.5-17.5 g/dL
Platelets	169	150-450 × 10^9^/L
Sodium	136	136-145 mmol/L
Potassium	4.1	3.5-5.5 mmol/L
Chloride	106	98-110 mmol/L
Bicarbonate	20	23-28 mmol/L
BUN	13	6-24 mg/dL
Creatinine	1.04	0.7-1.3 mg/dL
Glucose	301	70-140 mg/dL
Magnesium	2.3	1.5-2.5 mg/dL
Phosphorus	2.8	2.1-4.3 mg/dL
Troponin	4054	<34 ng/L
Lactic acid	5.9	0-2 mmol/L
Calcium	7.5	8.6-10.4 mg/dL
AST	44	10-36 U/L
ALT	35	10-49 U/L
ALP	39	46-116 U/L

The rest of the hospital course was uncomplicated. The patient was discharged on aspirin, metoprolol, atorvastatin, Plavix, and amiodarone, with outpatient follow-up with cardiology and cardiothoracic surgery.

## Discussion

Disorders of the cardiac conduction system pose a substantial risk to patients following cardiac surgery, and patients often require temporary pacing to prevent significant complications. Few studies have been done to highlight specific predictors for postoperative pacing [1,2]; however, these are outdated, and there is yet to be a general consensus on the criteria for selecting the use of epicardial pacing wires after cardiac surgery. A decision is usually made after weighing patient-specific risks and benefits and a multidisciplinary approach to decision-making by the cardiothoracic surgeons, anesthesiologists, and intensivists [3]. Epicardial pacing wires are crucial for early detection and suppression of atrial and ventricular arrhythmias, maintenance of an appropriate heart rate to enhance cardiac output, and optimization of hemodynamic status. Factors such as individual patient characteristics, staff experience and monitoring conditions, the complexity of the pacing system, and the frequency of performance checks all impact how the pacemaker system is programmed. 

The R-on-T phenomenon was first described by Smirk in 1949 as an R wave interrupting a T wave. It is essentially a type of PVC that can predispose to sustained tachyarrhythmia, including polymorphic VT and VF, which can lead to catastrophic complications, including death. Although it has been observed in patients with acute myocardial infarction [4] and in patients with long QT syndrome [5], there are only a few reported cases in settings of post-CABG epicardial pacing [6,7].

With temporary epicardial pacing, this phenomenon can occur when a random pacing signal is fired on a T wave while the intrinsic rhythm is occurring. This occurs due to the pacer wire's inability to sense appropriately, i.e., undersense the underlying cardiac electrical rhythm. Inappropriate stimulation during the cardiac cycle's repolarization phase can provoke life-threatening ventricular arrhythmias. Undersensing can happen as a result of improper lead positioning and high sensitivity values. Improper lead positioning is difficult to confirm once a patient is out of the operating room and is usually a postulated theory after sensing parameters are assessed and deemed accurate. The only solution is repositioning the wires, which would entail removing and replacing the wires altogether. Sensitivity refers to the minimum current an intrinsic signal has to hit before the pacemaker considers it an intrinsic rhythm. Essentially, it is the ability of a pacemaker to detect intrinsic cardiac activity. If the sensitivity value is too high, the pacemaker is considered less sensitive as it detects less cardiac activity. Conversely, a lower sensitivity value corresponds to greater sensitivity. If the pacemaker is checked and the sensitivity value is too high, it should be reduced [8-10]. Daily checks of pacemakers should be undertaken to reassess underlying rhythm, sensing, and capture thresholds. Epicardial wires usually tend to fail to sense and capture after four to five days and are only intended for short-term use for this reason. If pacing requirements persist, patients should be transitioned to permanent pacing. There is no consensus on the average length of time before inserting permanent pacemakers, but common practice ranges from 7 to 14 days [3,9].

Other risk factors for postoperative arrhythmias after cardiac surgery include patient-specific factors like advanced age, structural heart disease, and extracardiac comorbidities; and surgery-related factors including perioperative inflammation and trauma, ischemia, hypoxia, electrolyte imbalances, perioperative hemodynamic stress, and preoperative use of beta-blockers or digoxin [11]. Despite the absence of electrolyte imbalances, hypoxic or ischemic injury, and an appropriate duration of use of epicardial wires, our patient developed such a devastating complication. As there is currently no data in the literature to predict how epicardial wires will perform in the first few days of use, epicardial pacing failure is difficult to anticipate. Measures such as daily pacing checks, maintaining adequate electrolyte levels, maintaining adequate oxygenation, perfusion, and normothermia are some of the supportive strategies that can be employed to prevent postoperative arrhythmias in patients on temporary epicardial pacing.

## Conclusions

This case underscores the importance of the anticipation of postoperative arrhythmias in CABG patients on temporary epicardial pacing and the need to undertake preemptive measures to lower risks. As described in our case, the best strategy is to turn the pacemaker off or to remove the wires altogether if the risks outweigh the benefits.

## References

[1] Puskas JD, Sharoni E, Williams WH, Petersen R, Duke P, Guyton RA (2003). Is routine use of temporary epicardial pacing wires necessary after either OPCAB or conventional CABG/CPB?. Heart Surg Forum.

[2] Bethea BT, Salazar JD, Grega MA (2005). Determining the utility of temporary pacing wires after coronary artery bypass surgery. Ann Thorac Surg.

[3] Manuel L (2022). Temporary epicardial pacing wires post-cardiac surgery: a literature review. Gen Thorac Cardiovasc Surg.

[4] Chiladakis JA, Karapanos G, Davlouros P, Aggelopoulos G, Alexopoulos D, Manolis AS (2000). Significance of R-on-T phenomenon in early ventricular tachyarrhythmia susceptibility after acute myocardial infarction in the thrombolytic era. Am J Cardiol.

[5] Liu MB, Vandersickel N, Panfilov AV, Qu Z (2019). R-from-T as a common mechanism of arrhythmia initiation in long QT syndromes. Circ Arrhythm Electrophysiol.

[6] Nakamori Y, Maeda T, Ohnishi Y (2016). Reiterative ventricular fibrillation caused by R-on-T during temporary epicardial pacing: a case report. JA Clin Rep.

[7] Chemello D, Subramanian A, Kumaraswamy N (2010). Cardiac arrest caused by undersensing of a temporary epicardial pacemaker. Can J Cardiol.

[8] (2025). Temporary Pacing. https://www.cardioguide.ca/temporary-pacing/.

[9] Waqanivavalagi SW (2024). Temporary pacing following cardiac surgery - a reference guide for surgical teams. J Cardiothorac Surg.

[10] Reade MC (2007). Temporary epicardial pacing after cardiac surgery: a practical review: part 1: general considerations in the management of epicardial pacing. Anaesthesia.

[11] Peretto G, Durante A, Limite LR, Cianflone D (2014). Postoperative arrhythmias after cardiac surgery: incidence, risk factors, and therapeutic management. Cardiol Res Pract.

